# Epigenetic Modification Agents Improve Gene-Specific Methylation Reprogramming in Porcine Cloned Embryos

**DOI:** 10.1371/journal.pone.0129803

**Published:** 2015-06-11

**Authors:** Yanjun Huan, Zhanfeng Wu, Jiguang Zhang, Jiang Zhu, Zhonghua Liu, Xuexiong Song

**Affiliations:** 1 College of Animal Science and Technology, Qingdao Agricultural University, Qingdao, Shandong Province, China; 2 Shouguang City Hospital of Chinese Medicine, Weifang, Shandong Province, China; 3 College of Life Science, Northeast Agricultural University, Harbin, Heilongjiang Province, China; Guangzhou Institute of Biomedicine and Health, CHINA

## Abstract

Incomplete DNA methylation reprogramming in cloned embryos leads to poor cloning efficiency. Epigenetic modification agents can improve genomic methylation reprogramming and the development of cloned embryos, however, the effect of epigenetic modification agents on gene-specific methylation reprogramming remains poorly studied. Here, we investigated DNA methylation reprogramming of pluripotency (*Oct4*) and tissue specific (*Thy1*) genes during early embryo development in pigs. In this study, we found that compared with *in vitro* fertilized counterparts, cloned embryos displayed the disrupted patterns of *Oct4* demethylation and *Thy1* remethylation. When 5-aza-2'-deoxycytidine (5-aza-dC) or trichostatin A (TSA) enhanced the development of cloned embryos, the transcripts of *DNA methyltransferases* (*Dnmt1* and *Dnmt3a*), *histone acetyltransferase 1* (*Hat1*) and *histone deacetylase 1* (*Hdac1*) and the methylation and expression patterns of *Oct4* and *Thy1* became similar to those detected in *in vitro* fertilized counterparts. Further studies showed that *Dnmt1* knockdown in cloned embryos enhanced the methylation reprogramming of *Oct4* and *Thy1* and promoted the activation of *Oct4* and the silence of *Thy1*. In conclusion, our results demonstrated that cloned embryos displayed incomplete gene-specific methylation reprogramming and disrupted expression patterns of pluripotency and tissue specific genes, and epigenetic modification agents improved gene-specific methylation reprogramming and expression pattern by regulating epigenetic modification related genes. This work would have important implications in improving cloning efficiency.

## Introduction

Though somatic cell nuclear transfer (SCNT) has been achieved in many species, overall cloning efficiency is still low, and this limits the application of cloning technology in basic research, agriculture and medicine [1–3].

It is generally believed that low cloning efficiency is mainly due to aberrant epigenetic reprogramming [3]. During epigenetic reprogramming induced by SCNT, DNA methylation dynamics can reflect epigenetic reprogramming, therefore, the mechanism of epigenetic reprogramming in cloned embryos mainly focuses on DNA demethylation and remethylation [4]. After SCNT, DNA methylation reprogramming is usually incomplete, and this would cause no effective activation of pluripotency genes, continuous expression of tissue specific genes, aberrant transcription of imprinted genes, etc. in cloned embryos, thereby leading to poor cloning efficiency [3, 5–7].

To improve DNA methylation reprogramming and the development of cloned embryos, various strategies have been adopted, and the application of epigenetic modification agents enhances cloning efficiency [8–10]. Our previous studies also show that DNA methylation inhibitor (5-aza-dC) or histone deacetylase inhibitor (TSA) can improve genomic methylation reprogramming, the expression of genes related to early embryo development and the development of cloned embryos [11–13]. Thus, it is considered that gene-specific methylation reprogramming, referring to the erasure of donor cell original methylation status and the reestablishment of the methylation pattern required for embryo development, should be improved in these treated embryos. A previous study has shown that epigenetic modification agents can improve the methylation status of imprinted genes in cloned embryos [9], however, the effect of epigenetic modification agents on gene-specific methylation reprogramming in cloned embryos remains poorly studied.

To reveal the mechanism underlying the developmental improvement of cloned embryos treated with epigenetic modification agents, in this study, we investigated the methylation reprogramming of pluripotency (*Oct4*, a trigger for nuclear reprogramming) and tissue specific (*Thy1*, a fibroblast marker) genes during early embryo development [14, 15]. We identified the methylation regions of *Oct4* and *Thy1* promoters, and found that SCNT disrupted gene-specific methylation reprogramming in cloned embryos. When epigenetic modification agents enhanced the development of cloned embryos, gene-specific methylation reprogramming and expression pattern became similar to those in *in vitro* fertilized embryos. And more, *Dnmt1* knockdown also enhanced gene-specific methylation reprogramming. Thus, our results reveal that the improved gene-specific methylation reprogramming regulated by the appropriately corrected expression of epigenetic modification related genes is the mechanism underling the high development of cloned embryos treated with epigenetic modification agents, and this work would have important implications in improving cloning efficiency.

## Materials and Methods

Chemicals were purchased from Sigma-Aldrich Corporation (St. Louis, MO, USA), and disposable and sterile plasticware was obtained from Nunclon (Roskilde, Denmark), unless otherwise stated. All experiments were approved by the Animal Care Commission of Northeast Agricultural University according to animal welfare laws, guidelines and policies. All surgery was performed under sodium pentobarbital anaesthesia, and all efforts were made to minimize suffering.

### Porcine Fetal Fibroblasts (PFFs) Culture

PFFs culture has been described previously [12]. Briefly, porcine fetuses were obtained from a sow at day 35 of pregnancy after the sow was anaesthetized and sacrificed, then PFFs were isolated from 35-day-old fetuses under sodium pentobarbital anaesthesia. After removal of fetal head, internal organs and limbs, the remaining tissues were finely minced into pieces, digested with 0.25% trypsin-0.04% ethylenediaminetetraacetic acid solution (GIBCO), and then dispersed in high glucose enriched Dulbecco’s modified Eagle’s medium (DMEM, GIBCO) containing 10% fetal bovine serum (FBS, GIBCO) and 1% penicillin-streptomycin (GIBCO). The dispersed cells were centrifuged, resuspended and cultured in DMEM. Until confluence, PFFs were digested, centrifuged, resuspended in FBS containing 10% dimethyl sulfoxide and stored in liquid nitrogen until use. Prior to SCNT, PFFs were thawed, cultured and subsequently used in 3–5 passages.

### Oocyte Collection and In Vitro Maturation

Oocyte maturation has been described previously [12]. Briefly, porcine ovaries were collected from a slaughterhouse of Harbin Dazhong Roulian Food Co., Ltd., located in Harbin city, Heilongjiang province. Just after exposure, ovaries were placed in physiological saline with antibiotics at 37°C and transported to the laboratory. Follicles were aspirated, and follicular contents were washed with HEPES-buffered Tyrode's lactate. Cumulus-oocyte complexes (COCs) were recovered and cultured in maturation medium. After 42 h, COCs were vortexed in hyaluronidase for 30 sec to remove cumulus cells. Only oocytes with a visible polar body, regular morphology and a homogenous cytoplasm were used in the subsequent experiments.

### In Vitro Fertilization (IVF) and SCNT Embryo Culture and Treatment

The procedures for IVF and SCNT have been described [13, 16]. Briefly, for IVF, the semen was incubated and washed in DPBS supplemented with BSA. The spermatozoa were diluted with modified Tris-buffered medium (mTBM) to the appropriate concentration. Matured oocytes were washed in mTBM, transferred into fertilization medium and co-incubated with spermatozoa. Then, the embryos were washed and cultured in porcine zygote medium-3 (PZM-3) for subsequent development. For SCNT, matured oocytes and PFFs were placed into manipulation medium. After enucleation, donor cells were placed into the perivitelline space. Fusion and activation of the cell-cytoplast complexes were induced by electroporation. Then, reconstructed embryos were cultured in PZM-3 for subsequent development.

For 5-aza-dC or TSA treatment [13], cloned embryos were cultured in PZM-3 supplemented with 25 nM (optimized) 5-aza-dC (NT-AZA) or 40 nM (optimized) TSA (NT-TSA) for 24 h, then washed and transferred into PZM-3 for further culture.

### siRNA Design, Synthesis and Microinjection

According to the requirement of Invitrogen Block-iT RNAi Designer and the information of *Dnmt1* mRNA sequence, three Stealth siRNAs, related to *Dnmt1* conserved domains including the replication foci domain (RFD), bromo adjacent homology domain (BAH) and cytosine-C5 specific DNA methylase domain (DCM), were designed and synthesized (Invitrogen), and the sequences were as following: siRNA-RFD: CCCGTCTCTTGAAGGTGGTGTTAAT, siRNA-BAH: CATAGCAAAGTGAAGGTCATCTATA and siRNA-DCM: GATAAGAAGTTTGTCAGCAACATCA. Then, siRNAs were dissolved with Rnase free H_2_O to the concentration at 20 μM and microinjected into cloned embryos at 6 h post activation using Sterile Femtotips and the FemtoJet express microinjector (Eppendorf) [16]. The injection condition was 250 hpa Injection Pressure, 60 hpa Compensation Pressure and 0.7 sec Injection Time, and approximate 10 pl siRNAs were injected into cloned embryos. The same amount of negative siRNAs (NT-negative) was injected as the control.

For the detail procedure of embryo injection, cloned embryos were transferred into 200 μl drop of manipulation medium supplemented with 7.5 μg/ml cytochalasin B and 0.3% (w/v) BSA for microinjection. Immediately after microinjection, embryos were washed and cultured in PZM-3 for subsequent development. The interference efficiency was determined in 4-cell embryos, and the most effective interference sequence (NT-siRNA) was applied in the subsequent experiments.

### Embryo Collection

For embryo collection, 1-cell, 2-cell, 4-cell, 8-cell and blastocyst embryos in the IVF, NT-CON (cloned), NT-AZA, NT-TSA, NT-negative and NT-siRNA groups were collected at 6 h, 24 h, 48 h, 72 h and 156 h, respectively.

### Bisulfite Sequencing

Bisulfite sequencing has been reported [13]. Briefly, pooled samples were digested with Proteinase K (PK) and treated with sodium bisulfite to convert all unmethylated cytosine to uracil using an EZ DNA Methylation-Direct Kit (Zymo Research). For semen, the sperm was collected by centrifugation, washed in SMB solution (10 mM Tris-HCl, 10 mM EDTA, 50 mM NaCl and 2% SDS) and incubated in SMB solution supplemented with 40 mM dithiothreitol and 0.3 mg/ml PK at 56°C for 1 h. For samples of 10^3^ PFFs, 200 MII oocytes and 200, 100, 50, 25 and 20 pooled zona pellucida-removed embryos at the 1-cell, 2-cell, 4-cell, 8-cell and blastocyst stages, respectively, digestion was performed in M-Digestion Buffer supplemented with PK at 50°C for 20 min. After digestion, a CT (cytosine to thymine) conversion reagent was added at 98°C for 10 min and 64°C for 2.5 h. Then, the samples were desalted, purified and diluted with M-Elution Buffer. Subsequently, nested PCR was carried out to amplify the target regions of Oct4 and Thy1 using the primers described in S1 Table and Hot Start Taq Polymerase (TaKaRa) with a profile of 94°C for 5 min, 45 cycles of 94°C for 30 sec, the optimal annealing temperature (53°C for Oct4 Region I, 54°C for Oct4 Region II, 50°C for Thy1 Regions I and II, and 53°C for Thy1 Region III, respectively) for 30 sec and 72°C for 1 min, followed by 72°C for 10 min. Products from the first amplification reaction were used in the second PCR reaction, and the optimal annealing temperatures of inner primers were 50°C for Oct4 Regions I and II, 51°C for Thy1 Regions I and II and 52°C for Thy1 Region III. Then, the amplified products were verified by electrophoresis and purified using an Agarose Gel DNA Purification Kit (TaKaRa). The purified fragments were cloned into pMD18-T Vectors (TaKaRa) and subjected to sequence analysis.

### Quantitative Real-Time PCR

Measurement of gene expression with quantitative real-time PCR has been applied [12, 16]. Briefly, total RNA was extracted from 50 pooled embryos at each stage using an RNeasy Mini Kit (Qiagen) according to the manufacturer’s instructions, and the elution volume was 50 μl. Reverse transcription was performed using a PrimeScript RT Reagent Kit (TaKaRa). The 100 μl reaction volume contained 20 μl 5×PrimeScript Buffer, 5 μl PrimeScript RT Enzyme Mix I, 5 μl Oligo dT Primer (50 μM), 5 μl Random 6 mers (100 μM), 50 μl Total RNA, and 15 μl RNase Free dH_2_O. The reaction was 37°C for 15 min and 85°C for 5 sec, and the cDNA was stored at -20°C until use. For quantitative real-time PCR, reactions were performed in 96-well optical reaction plates (Applied Biosystems) using SYBR Premix ExTaq II (TaKaRa) and a 7500 Real-Time PCR System (Applied Biosystems). Each reaction mixture (20 μl) contained 2 μl cDNA solution, 10 μl 2×SYBR *Premix Ex Taq* II, 1.6 μl PCR primer (10 μM), 0.4 μl ROX Reference Dye II (50×) and 6 μl dH_2_O. Thermal cycling conditions were 95°C for 30 sec, followed by 40 two-step cycles of 95°C for 5 sec and 60°C for 34 sec and finally a dissociation stage consisting of 95°C for 15 sec, 60°C for 1 min and 95°C for 15 sec. For each sample, the cycle threshold (CT) values were obtained from three replicates. The primers used for amplification of target and internal reference genes were presented in S1 Table. The relative expression levels of target genes were analyzed using the 2^−ΔΔCT^ method.

### Statistical Analysis

Differences in data (mean ± SEM) were analyzed with the SPSS statistical software. Statistical analysis of data concerning DNA methylation, gene expression and embryo development were performed with one-way analysis of variance. For all analyses, differences were considered to be statistically significant when P<0.05.

## Results

### Incomplete Gene-Specific Methylation Reprogramming in Porcine Cloned Embryos

To examine gene-specific methylation dynamics in early embryos, we analyzed the distribution of CpG sites in *Oct4* and *Thy1* promoters using the MethPrimer program, and observed 2 CpG islands in *Oct4* promoter and 1 CpG island in *Thy1* promoter, respectively (S1 Fig). Then, the expression patterns of *Oct4* and *Thy1* and the methylation statuses of different regions of *Oct4* (Regions I and II) and *Thy1* (Regions I, II and III) promoters in sperms, oocytes, PFFs and IVF blastocysts were examined (S1 Fig). We detected the relative expression of *Oct4* in oocytes and blastocysts and *Thy1* in PFFs, and found that oocyte or blastocyst methylation level was significantly lower than those of sperms and PFFs in Region I of *Oct4* promoter and not significantly different from that of sperms in Region II of *Oct4* promoter (P<0.05), and PFF methylation level displayed no significant difference from that of oocytes in Region I of *Thy1* promoter but significant differences from those of sperms, oocytes and blastocysts in Regions II and III of *Thy1* promoter (P<0.05), and was even lower in Region II than Region III of *Thy1* promoter, suggesting that Region I of *Oct4* promoter, including 16 CpG sites or Region II (10 CpG sites) of *Thy1* promoter could represent the methylation status of *Oct4* or *Thy1*, respectively.

**Fig 1:**
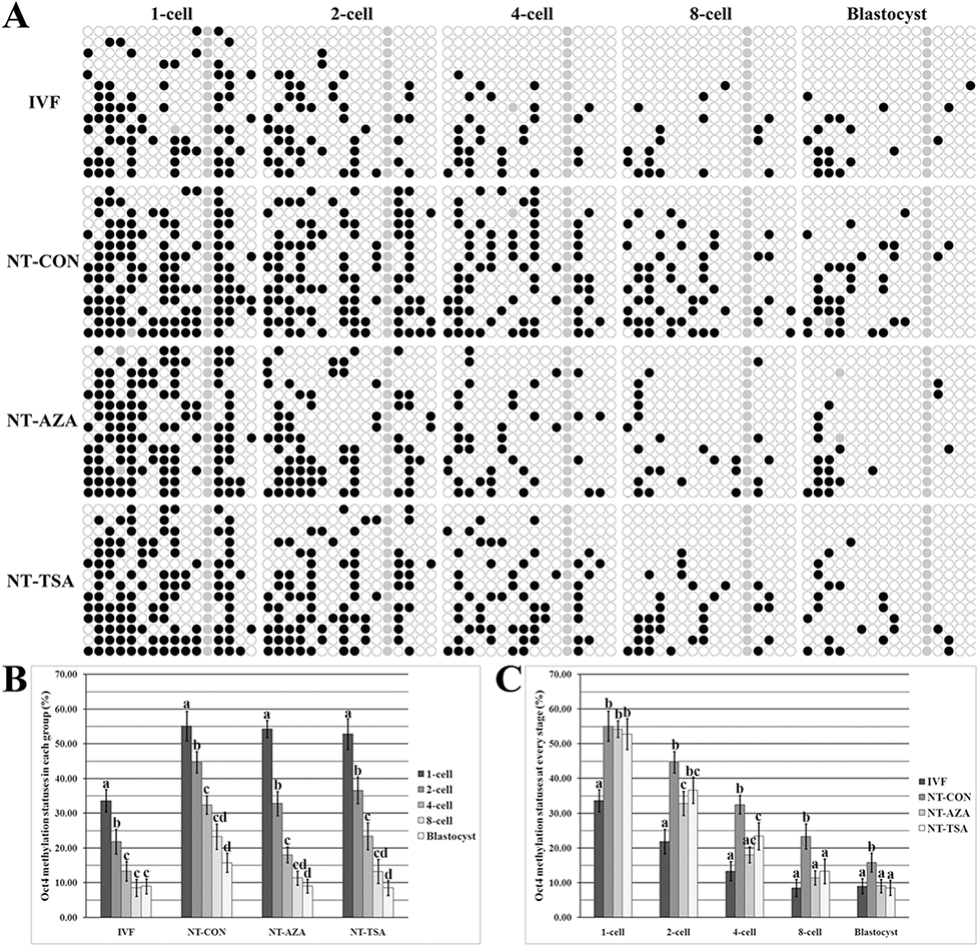
Oct4 methylation statuses in early embryos. A, the methylation statuses of Oct4 at 1-cell, 2-cell, 4-cell, 8-cell and blastocyst stages of IVF, NT-CON, NT-AZA and NT-TSA embryos, B, the methylation levels of Oct4 in the IVF, NT-CON, NT-AZA and NT-TSA groups, and C, the methylation levels of Oct4 at different stages of IVF, NT-CON, NT-AZA and NT-TSA embryos. Cloned embryos displayed incomplete methylation reprogramming of Oct4, while 5-aza-dC or TSA rescued the disrupted methylation pattern of Oct4 in cloned embryos. Black or white circles indicate methylated or unmethylated CpG sites, respectively, and gray circles represent mutated and/or single nucleotide polymorphism (SNP) variation at certain CpG sites. ^a-d^Values in the same group or at a given stage with different superscripts differ significantly (P<0.05).

**Fig 2:**
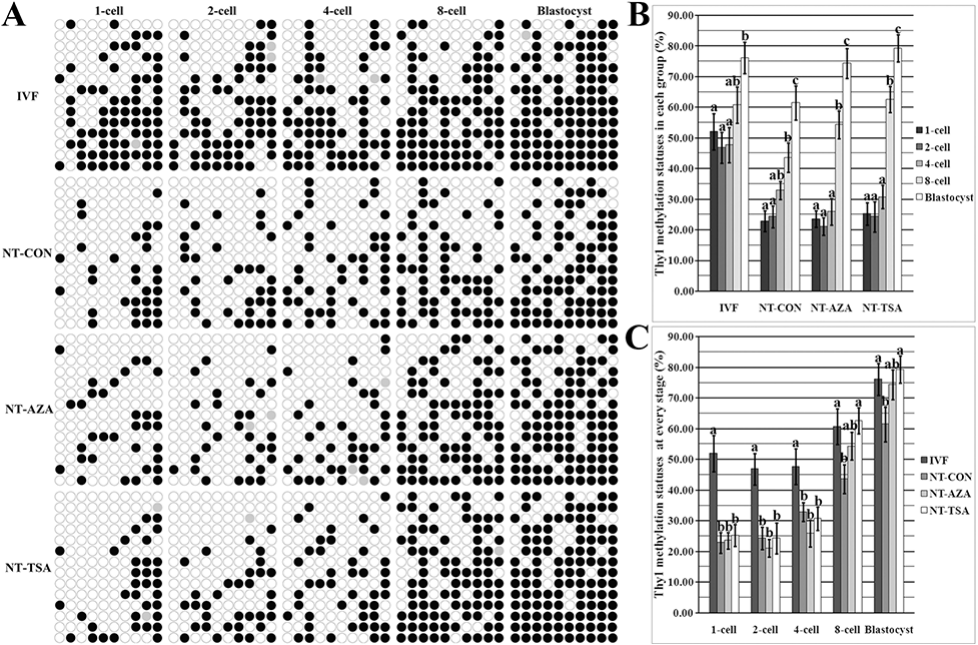
Thy1 methylation statuses in early embryos. A, the methylation statuses of Thy1 at 1-cell, 2-cell, 4-cell, 8-cell and blastocyst stages of IVF, NT-CON, NT-AZA and NT-TSA embryos, B, the methylation levels of Thy1 in the IVF, NT-CON, NT-AZA and NT-TSA groups, and C, the methylation levels of Thy1 at different stages of IVF, NT-CON, NT-AZA and NT-TSA embryos. Cloned embryos displayed the disrupted methylation pattern of Thy1, while 5-aza-dC or TSA promoted Thy1 remethylation in cloned embryos. Black or white circles indicate methylated or unmethylated CpG sites, respectively, and gray circles represent mutated and/or SNP variation at certain CpG sites. ^a-c^Values in the same group or at a given stage with different superscripts differ significantly (P<0.05).

Then, the methylation statuses of *Oct4* and *Thy1* were examined in IVF embryos (Figs 1 and 2). *Oct4* showed a gradual and significant demethylation from the 1-cell to 4-cell stage (P<0.05) and maintained a low methylation status from the 4-cell to blastocyst stage, while the methylation pattern of *Thy1* took on an upward trend from the 2-cell to blastocyst stage, and the methylation level of *Thy1* in blastocysts was significantly higher that those in 2-cell and 4-cell embryos (P<0.05). Thus, IVF embryos displayed a low methylation status of *Oct4* and a high methylation level of *Thy1*.

After SCNT (Figs 1 and 2), significantly reduced methylation levels of *Oct4* were observed in cloned embryos (2-cell vs 1-cell, 4-cell vs 2-cell and blastocyst vs 4-cell, respectively, P<0.05), and for *Thy1*, the methylation level in 8-cell or blastocyst embryos was significantly (P<0.05) higher than that in 2-cell or 8-cell embryos, respectively. Thus, cloned embryos also displayed the gradual demethylation of *Oct4* and remethylation of *Thy1*. When comparing the individual developmental stage between cloned and IVF embryos, we found that, compared with those in IVF counterparts, cloned embryos displayed significantly higher methylation levels of *Oct4* and lower methylation statuses of *Thy1* at all the stages (P<0.05). These results suggested that gene-specific methylation reprogramming was incomplete in porcine cloned embryos.

### Epigenetic Modification Agents Improved Gene-Specific Methylation Reprogramming in Porcine Cloned Embryos

After cloned embryos treated with 5-aza-dC or TSA, the development of cloned embryos was significantly enhanced and the expression levels of *Dnmt1*, *Dnmt3a*, *Hat1*, and *Hdac1* became similar to those in IVF embryos (S2 and S3 Figs and Table 1), suggesting that the methylation patterns of *Oct4* and *Thy1* in these treated embryos should be ameliorated (Figs 1 and 2).

**Table 1:** Development of cloned embryos treated with 5-aza-dC or TSA.

Groups	No. embryos (Rep.)	No. embryos cleaved (% ± SEM)	No. blastocysts (% ± SEM)
**NT-CON**	131 (3)	114 (87.11 ± 0.70)^a^	27 (20.76 ± 1.19)^a^
**NT-AZA**	128 (3)	113 (88.26 ± 0.32)^a^ ^b^	35 (27.36 ± 0.61)^b^
**NT-TSA**	126 (3)	113 (89.68 ± 0.80)^b^	63 (49.98 ± 2.46)^c^

Treating cloned embryos with 25 nM 5-aza-dC or 40 nM TSA for 24 h enhanced the cleavage and blastocyst rates of cloned embryos.

^a-c^Values in the same column with different superscripts differ significantly (P<0.05).

In the NT-AZA group, *Oct4* underwent a similar demethylation trend to that in the NT-CON group, and *Thy1* displayed a gradual remethylation from the 2-cell to blastocyst stage, with the significantly higher methylation level at the 8-cell or blastocyst stage than the 4-cell or 8-cell stage, respectively (P<0.05). In comparison with the NT-CON group, the NT-AZA group showed significantly lower methylation levels of *Oct4* from the 2-cell to blastocyst stage (P<0.05) and higher *Thy1* methylation levels in 8-cell and blastocyst embryos. And, no significant differences of *Oct4* methylation statuses from the 4-cell to blastocyst stage and *Thy1* methylation levels in 8-cell and blastocyst embryos were observed between the NT-AZA and IVF groups, though 1-cell and 2-cell embryos still displayed significant differences (P<0.05). Thus, 5-aza-dC improved the methylation reprogramming of *Oct4* and *Thy1* in porcine cloned embryos.

After TSA treatment, the trends of *Oct4* demethylation and *Thy1* remethylation were similar to those in the NT-AZA group, and no significant differences of *Oct4* and *Thy1* methylation levels were observed between the NT-TSA and NT-AZA groups. When compared with the NT-CON group, the NT-TSA group showed significantly lower methylation levels of *Oct4* from the 4-cell to blastocyst stage and higher *Thy1* methylation levels in 8-cell and blastocyst embryos (P<0.05). And, there were no significant differences of *Oct4* and *Thy1* methylation levels at the 8-cell and blastocyst stages between the NT-TSA and IVF groups. Thus, TSA also improved *Oct4* and *Thy1* methylation reprogramming in porcine cloned embryos. Overall, these results suggested that the epigenetic modification agent 5-aza-dC or TSA improved gene-specific methylation reprogramming in porcine cloned embryos.

### Epigenetic Modification Agents Promoted the Activation of Pluripotency Genes and the Silence of Tissue Specific Genes in Porcine Cloned Embryos

**Fig 3:**
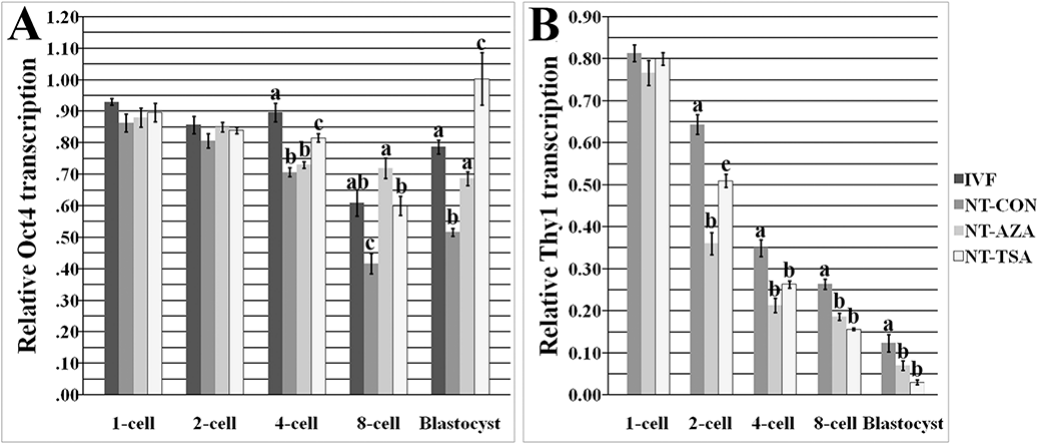
Relative Oct4 and Thy1 transcripts in early embryos. A and B, relative transcripts of Oct4 and Thy1 at 1-cell, 2-cell, 4-cell, 8-cell and blastocyst stages of IVF, NT-CON, NT-AZA and NT-TSA embryos, respectively. 5-aza-dC or TSA appropriately promoted the expression of Oct4 and silenced the transcription of Thy1 in cloned embryos. The transcript abundance in MII oocytes (A) or cloned embryos at 2 h post activation (B) was considered to be the control. The data were expressed as mean ± SEM. ^a-c^Values at a given stage for the same gene with different superscripts differ significantly (P<0.05).

Generally, the improvement of DNA methylation reprogramming would effectively regulate gene expression [17]. Thus, the enhanced methylation reprogramming of *Oct4* and *Thy1* induced by 5-aza-dC or TSA should lead to their appropriate expression (Fig 3).

In IVF embryos, *Oct4* expression was downward from the 1-cell to 8-cell stage, and slightly upregulated at the blastocyst stage. In cloned embryos, *Oct4* took on a similar transcription pattern to that in IVF embryos, but the expression levels from the 4-cell to blastocyst stage were significantly lower (P<0.05), and, *Thy1* transcripts displayed a gradual decline. After 5-aza-dC treatment, compared with the NT-CON group, the NT-AZA group showed significantly higher transcripts of *Oct4* in 8-cell and blastocyst embryos and lower expression of *Thy1* from the 2-cell to blastocyst stage (P<0.05), and the expression levels of *Oct4* in 8-cell and blastocyst embryos were not significantly different from those in IVF embryos. In the NT-TSA group, significantly upregulated *Oct4* transcripts from the 4-cell to blastocyst stage and downregulated *Thy1* expression from the 2-cell to blastocyst stage were observed in comparison with the NT-CON group (P<0.05), and the NT-TSA group even showed a significant mRNA increase of *Oct4* at the blastocyst stage compared with the IVF group, though a significant reduction at the 4-cell stage was still observed (P<0.05). When the individual developmental stage between the NT-TSA and NT-AZA groups was compared, the overall expression patterns of *Oct4* and *Thy1* were similar, except that significantly higher transcription levels of *Oct4* at the 4-cell and blastocyst stages and *Thy1* at the 2-cell stage and a significant reduction of *Oct4* expression at the 8-cell stage were observed in the NT-TSA group (P<0.05). And more, 5-aza-dC or TSA significantly promoted the expression of *Nanog* and *Sox2* and the silence of *Col5a2* in cloned embryos (S4 Fig, P<0.05). Thus, these results showed that the expression patterns of pluripotency and tissue specific genes were disrupted in cloned embryos, and the epigenetic modification agent 5-aza-dC or TSA effectively promoted the activation of pluripotency genes and the silence of tissue specific genes in porcine cloned embryos.

### *Dnmt1* Knockdown Improved Gene-Specific Methylation Reprogramming in Porcine Cloned Embryos

To further explore the mechanism underlying the improvement of gene-specific methylation reprogramming induced by 5-aza-dC or TSA, RNA interference (RNAi) was applied to reduce *Dnmt1* expression in cloned embryos, as both the NT-AZA and NT-TSA groups showed significantly lower transcripts of *Dnmt1* in comparison with the NT-CON group (S3 Fig). When siRNAs targeting *Dnmt1* were injected into cloned embryos, compared with the controls noninjected or injected with negative siRNAs, siRNA-RFD, siRNA-BAH and siRNA-DCM resulted in significant reductions (74.45%, 79.69% and 83.42%, respectively) in *Dnmt1* transcription at the 4-cell stage (S5 Fig, P<0.05). Thus, RNAi could significantly reduce *Dnmt1* expression, and siRNA-DCM was the most effective interference sequence. After siRNA-DCM injection (the NT-siRNA group), the blastocyst rate was significantly higher than that in the NT-CON or NT-negative group (S6 Fig and Table 2).

**Table 2:** Development of cloned embryos after Dnmt1 knockdown.

Groups	No. embryos (Rep.)	No. embryos cleaved (% ± SEM)	No. blastocysts (% ± SEM)
**NT-CON**	127 (3)	111 (87.43 ± 0.38)	25 (19.75 ± 1.21)^a^
**NT-negative**	121 (3)	106 (87.63 ± 1.06)	24 (19.86 ± 1.06)^a^
**NT-siRNA**	123 (3)	109 (88.36 ± 1.64)	32 (26.05 ± 0.53)^b^

Dnmt1 knockdown in cloned embryos at 6 h post activation improved the blastocyst rate.

^a-b^Values in the same column with different superscripts differ significantly (P<0.05).

**Fig 4:**
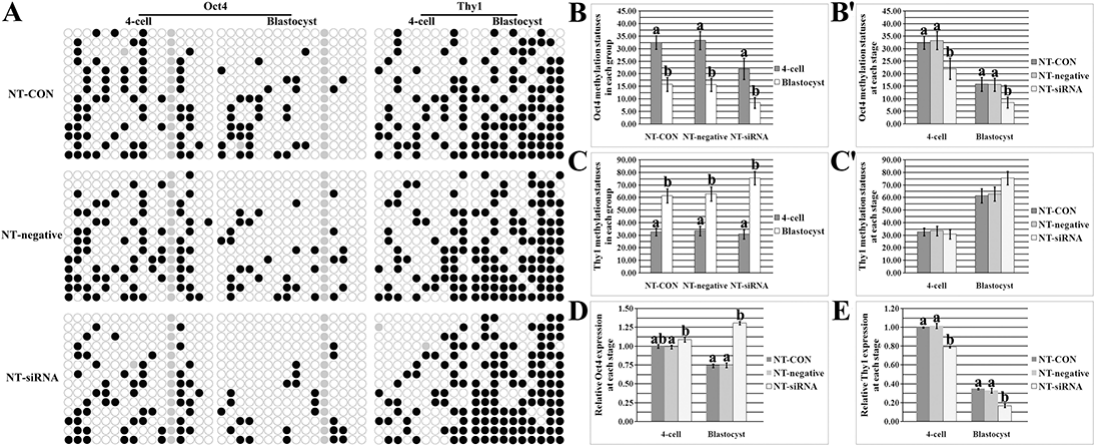
The methylation and expression patterns of Oct4 and Thy1 in cloned embryos after Dnmt1 knockdown. A, the methylation statuses of Oct4 and Thy1 at the 4-cell and blastocyst stages of NT-CON, NT-negative and NT-siRNA embryos, B and B', the methylation levels of Oct4 at the 4-cell and blastocyst stages of NT-CON, NT-negative and NT-siRNA embryos, C and C', the methylation levels of Thy1 at the 4-cell and blastocyst stages of NT-CON, NT-negative and NT-siRNA embryos, D and E, relative transcripts of Oct4 and Thy1 at the 4-cell and blastocyst stages of NT-CON, NT-negative and NT-siRNA embryos, respectively. Dnmt1 knockdown improved the methylation reprogramming of Oct4 and Thy1 and promoted the activation of Oct4 and the silence of Thy1 in cloned embryos. The transcript abundance of Oct4 or Thy1 in 4-cell cloned embryos was considered to be the control. The data were expressed as mean ± SEM. ^a-b^Values at a given stage or group in the same column chart with different superscripts differ significantly (P<0.05).

After *Dnmt1* knockdown (Fig 4), compared with the NT-CON or NT-negative group, the NT-siRNA group displayed significantly reduced methylation levels of *Oct4* at the 4-cell and blastocyst stages (P<0.05) and a higher *Thy1* methylation level in blastocysts, and significantly upregulated transcripts of *Oct4* and significant reductions in *Thy1* expression at the 4-cell and blastocyst stages were also observed in the NT-siRNA group (P<0.05). And more, *Dnmt1* knockdown significantly increased the expression levels of *Dnmt1*, *Dnmt3a*, *Hat1*, *Hdac1*, *Nanog* and *Sox2* and reduced the transcription of *Col5a2* in cloned blastocysts (S7 Fig, P<0.05). Thus, these results showed that *Dnmt1* knockdown improved the methylation reprogramming of pluripotency and tissue specific genes in cloned embryos, suggesting that the mechanism underlying the improvement of gene-specific methylation reprogramming induced by epigenetic modification agents could be the appropriately corrected expression patterns of genes related to epigenetic modification.

## Discussion

**Fig 5:**
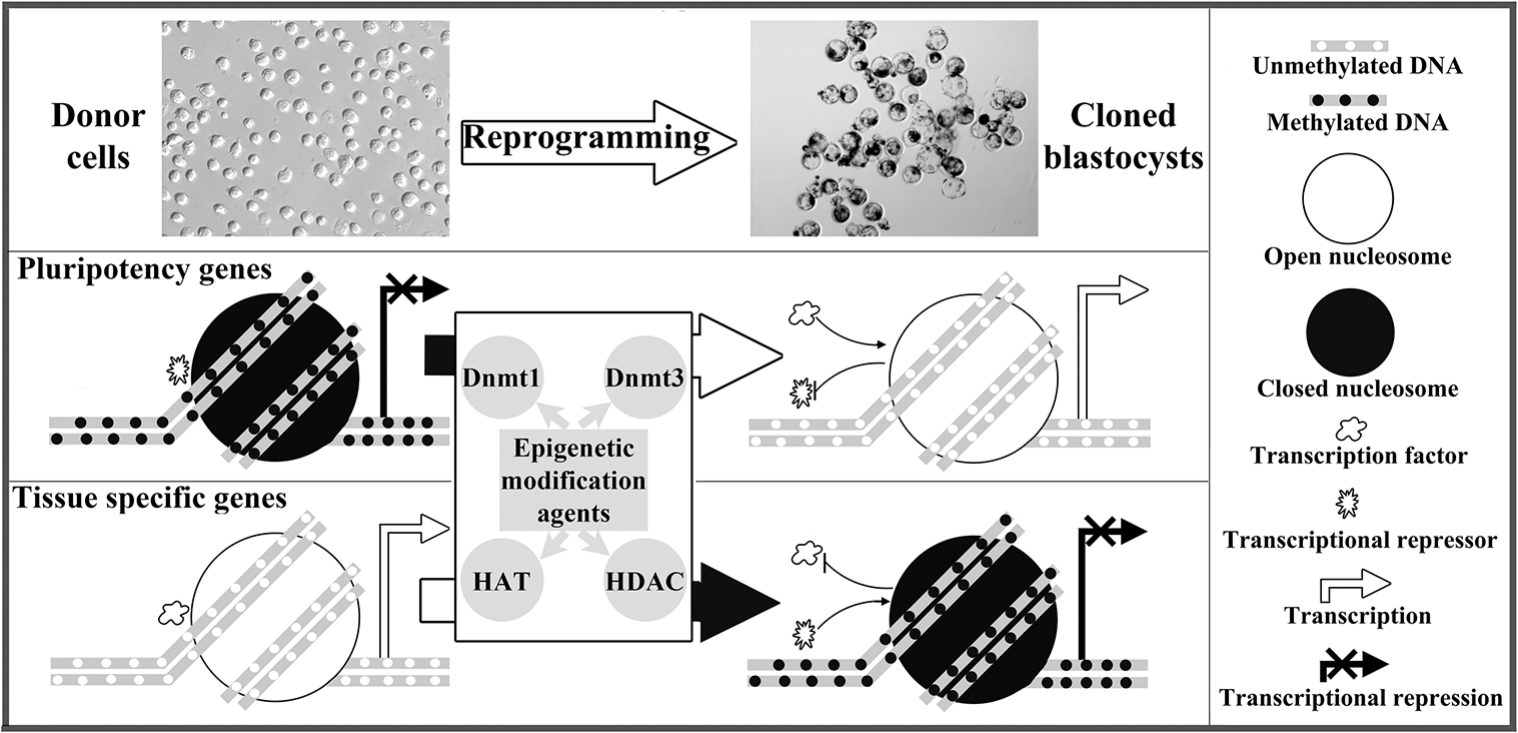
The potential mechanism of gene-specific methylation reprogramming induced by epigenetic modification agents in cloned embryos. 5-aza-dC or TSA improved the methylation reprogramming of pluripotency and tissue specific genes by regulating the expression of epigenetic modification related genes in cloned embryos.

Epigenetic modification agents can enhance the development of cloned embryos [11–13]. In this study, we demonstrated that 5-aza-dC or TSA improved gene-specific methylation reprogramming, and effectively promoted the activation of pluripotency genes and the silence of tissue specific genes in porcine cloned embryos, and the mechanism underlying the improvement of cloned embryo development and gene-specific methylation reprogramming induced by epigenetic modification agents could be the appropriately corrected expression of epigenetic modification related genes (Fig 5).

DNA methylation reprogramming in cloned embryos refers to the erasure of donor cell original methylation status and the reestablishment of embryonic methylation characteristics [7, 18, 19]. Previous studies have shown that genomic methylation reprogramming is aberrant in cloned embryos [3, 7, 20]. In this study, pluripotency and tissue specific genes were selected to study DNA methylation reprogramming, as the methylation patterns of these genes can reflect the epigenetic reprogramming degree in cloned embryos[14, 21]. Generally, the effective demethylation of pluripotency genes and remethylation of tissue specific genes are necessary for the normal development of cloned embryos [18]. However, in this study, gene-specific methylation reprogramming was incomplete in porcine cloned embryos, which probably leads to poor development of cloned embryos. As for the reason for incomplete methylation reprogramming of *Oct4* and *Thy1* in cloned embryos, it is possible that there is a potential mechanism which preserves the tissue specific methylation pattern of donor cells against reprogramming by oocyte factors [13, 22, 23].

It is known that epigenetic modification agents can regulate DNA methylation [13], thus, along with the high development of cloned embryos treated with epigenetic modification agents, DNA methylation reprogramming should be improved in these treated embryos. In this study, our results were consistent with this hypothesis, showing that gene-specific methylation reprogramming was improved in cloned embryos after 5-aza-dC or TSA treatment, similar to the pattern in IVF embryos. This facilitated methylation reprogramming of *Oct4* and *Thy1* induced by 5-aza-dC or TSA further led to their appropriate expression. Previous studies have reported that the appropriate activation of pluripotent genes and silence of tissue specific genes are required for the normal development of cloned embryos [3, 18, 19]. Thus, the developmental improvement of cloned embryos after 5-aza-dC or TSA treatment was probably due to that the rescued gene-specific methylation reprogramming enhanced the restoration of the expression patterns of genes related to the development of cloned embryos.

During DNA methylation reprogramming in early embryos, our results displayed that *Oct4* took on a demethylation pattern, while *Thy1* showed a remethylation trend, reflecting the diversity of gene-specific methylation reprogramming. And, in IVF embryos, the methylation patterns of different genes are also reported to be various [24]. Thus, the different methylation dynamics of certain genes are essential for the normal development of early embryos, and the regulation manner of gene-specific methylation reprogramming could be different. And, the enhanced gene-specific methylation reprogramming after 5-aza-dC or TSA treatment could be due to the improvement of different regulation manners. As for the improvement of *Oct4* demethylation in 5-aza-dC or TSA treated embryos, it is possibly due to that 5-aza-dC was incorporated into genome during DNA replication, or TSA loosened chromatin structure, beneficial for the binding of DNA demethylation related factors, and the reduced expression of *Dnmt1* and *Hdac1* and the upregulated *Hat1* transcription could be also the cause [1, 13, 20]. And, for the effective *Thy1* remethylation after 5-aza-dC or TSA treatment, the upregulated expression of *Dnmt3a* and *Hdac1* after zygote genome activation could be the potential cause, as *Dnmt3a* carries out de novo methylation and *Hdac1* can make chromatin condensation, leading to gene silencing and methylation [25–27]. These two different gene-specific methylation reprogramming and potential regulation patterns suggested that genes regulating DNA methylation reprogramming can selectively modify the methylation patterns of early embryo development related genes according to the developmental requirement. As for the detail mechanism of gene-specific methylation reprogramming in cloned embryos and how epigenetic modification agents improved this mechanism, further investigation is needed.

Generally, the improvement of DNA methylation reprogramming in cloned embryos induced by epigenetic modification agents is considered to be through the appropriate adjustment of the expression patterns of epigenetic modification related genes [9, 28, 29]. After cloned embryo treatment with epigenetic modification agents, our results showed that the expression patterns of epigenetic modification related genes, especially *Dnmt1*, were similar to those in IVF embryos, indicating that the disrupted expression pattern of *Dnmt1* could be a potential factor restricting gene-specific methylation reprogramming in cloned embryos. A previous study has shown that *Dnmt1* knockdown improves the development of cloned embryos [23]. Here, *Dnmt1* knockdown enhanced gene-specific methylation reprogramming and the development of cloned embryos. Thus, the appropriate expression of *Dnmt1* induced by epigenetic modification agents could be one mechanism underlying the improvement of gene-specific methylation reprogramming, further leading to the high development of cloned embryos. As for the improved gene-specific methylation reprogramming after *Dnmt1* knockdown, the reduced expression of *Dnmt1* in 4-cell cloned embryos and the increased transcripts of epigenetic modification related genes in cloned blastocysts could be the possible reason. Certainly, other molecules also participate in gene-specific methylation reprogramming [26, 30, 31], thus, more studies are still needed to reveal the detail mechanism of gene-specific methylation reprogramming in cloned embryos.

During gene-specific methylation reprogramming, 5-hydroxymethylcytosine should also play a key role [32], even though traditional bisulfite sequencing could not distinguish 5-hydroxymethylcytosine from 5-methylcytosine [33]. In view of the critical role of 5-hydroxymethylcytosine in nuclear reprogramming, new technologies such as oxidative bisulfite sequencing would be employed to investigate gene-specific methylation reprogramming in the further research.

In conclusion, our results showed that gene-specific methylation reprogramming was incomplete in porcine cloned embryos, and treating cloned embryos with 5-aza-dC or TSA improved gene-specific methylation reprogramming and expression pattern. And more, *Dnmt1* knockdown also enhanced gene-specific methylation reprogramming. Thus, the improved gene-specific methylation reprogramming and expression pattern regulated by the appropriately corrected expression of epigenetic modification related genes induced by epigenetic modification agents resulted in the high development of cloned embryos.

## Supporting Information

S1 Fig Prediction and analysis of Oct4 and Thy1 methylation statuses. A, prediction and analysis of Oct4 methylation statuses in sperms, oocytes, PFFs and blastocysts. 30 CpG sites (16 in Region I and 14 in Region II, respectively) were analyzed in Oct4 sequence around ATG by the MethPrimer program. B, prediction and analysis of Thy1 methylation statuses in sperms, oocytes, PFFs and blastocysts. 34 CpG sites (17 in Region I, 10 in Region II and 7 in Region III, respectively) were analyzed in Thy1 sequence around ATG by the MethPrimer program. C, the methylation levels of different regions of Oct4 and Thy1 in sperms, oocytes, PFFs and IVF blastocysts. According to the expression levels and methylation patterns of Oct4 and Thy1 in sperms, oocytes, PFFs and blastocysts, Region I of Oct4, including 16 CpG sites or Region II (10 CpG sites) of Thy1 could represent the methylation status of Oct4 or Thy1, respectively. Black or white circles indicate methylated or unmethylated CpG sites, respectively, and gray circles represent mutated and/or SNP variation at certain CpG sites. (+) represents gene expression, while (-) stands for no expression. The data are expressed as means ± SEM. ^a-d^Values in the same group or at a given stage with different superscripts differ significantly (P<0.05).
(TIF)

S2 Fig Blastocysts of cloned embryos. A, B and C, blastocysts (×40) derived from cloned embryos untreated, treated with 25 nM 5-aza-dC and treated with 40 nM TSA, respectively.
(TIF)

S3 Fig Relative transcripts of DNA methylation and histone acetylation related genes in early embryos. The expression patterns of Dnmt1 (A), Dnmt3a (B), Hat1 (C) and Hdac1 (D) at the 1-cell, 2-cell, 4-cell, 8-cell and blastocyst stages of IVF, NT-CON, NT-AZA and NT-TSA embryos. In comparison with IVF embryos, cloned embryos displayed the disrupted expression patterns of Dnmt1, Dnmt3a, Hat1 and Hdac1, while the expression profiles of these genes in NT-AZA or NT-TSA embryos were appropriately adjusted. The transcript abundance in MII oocytes was considered to be the control. The data were expressed as mean ± SEM. ^a-d^Values with different superscripts differ significantly (P<0.05).
(TIF)

S4 Fig Relative transcripts of Nanog, Sox2 and Col5a2 in early embryos. Relative transcripts of Nanog (A), Sox2 (B) and Col5a2 (C) at the 4-cell and blastocyst stages of IVF, NT-CON, NT-AZA and NT-TSA embryos. 5-aza-dC or TSA promoted the expression of Nanog, Sox2 and silenced the transcription of Col5a2 in cloned embryos. The transcript abundance in 4-cell cloned embryos was considered to be the control. The data were expressed as mean ± SEM. ^a-d^Values at a given stage for the same gene with different superscripts differ significantly (P<0.05).
(TIF)

S5 Fig The interference efficiencies of different siRNAs. After siRNAs were injected into cloned embryos at 6 h post activation, the interference efficiency was measured in 4-cell cloned embryos. siRNA-RFD, siRNA-BAH and siRNA-DCM significantly reduced the expression of Dnmt1, and the interference efficiency of siRNA-DCM was the highest. The data were expressed as mean ± SEM. ^a-c^Values with different superscripts differed significantly (P<0.05).
(TIF)

S6 Fig Blastocysts of cloned embryos after siRNA injection. A, B and C, blastocysts (×40) derived from cloned embryos untreated, injected with negative siRNA and injected with siRNA.
(TIF)

S7 Fig Relative transcripts of Dnmt1, Dnmt3a, Hat1, Hdac1, Nanog, Sox2 and Col5a2 in blastocysts after Dnmt1 knockdown. Compared with the NT-CON or NT-negative group, the NT-siRNA group displayed the upregulated expression of Dnmt1, Dnmt3a, Hat1, Hdac1, Nanog, Sox2 and downregulated transcription of Col5a2 in blastocysts. The transcript abundance of each gene in NT-CON embryos was considered to be the control. The data were expressed as mean ± SEM. ^a-b^Values at a given stage for the same gene with different superscripts differ significantly (P<0.05).
(TIF)

S1 Table PCR primers. The primer sequence, amplified length and gene accession number for bisulfite sequencing and quantitative real-time PCR.
(PDF)
