# Supplementary figures and images for: Epigenetic Modification Agents Improve Gene-Specific Methylation Reprogramming in Porcine Cloned Embryos
Source: PLoS One. 2015 Jun 11;10(6):e0129803. doi: 10.1371/journal.pone.0129803 (PMC4465902; doi:10.1371/journal.pone.0129803)

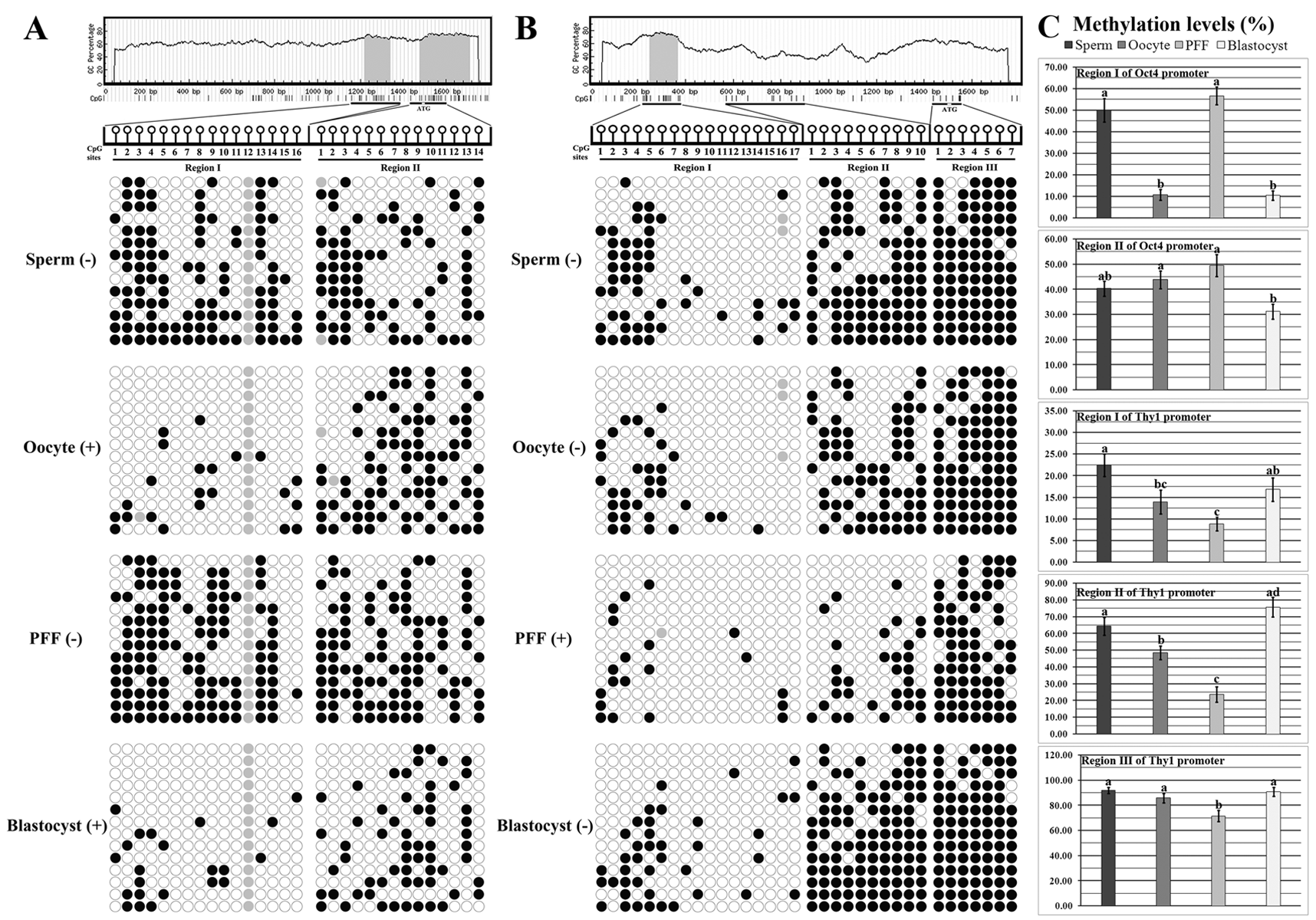

Supplement: S1 Fig — A, prediction and analysis of Oct4 methylation statuses in sperms, oocytes, PFFs and blastocysts. 30 CpG sites (16 in Region I and 14 in Region II, respectively) were analyzed in Oct4 sequence around ATG by the MethPrimer program. B, prediction and analysis of Thy1 methylation statuses in sperms, oocytes, PFFs and blastocysts. 34 CpG sites (17 in Region I, 10 in Region II and 7 in Region III, respectively) were analyzed in Thy1 sequence around ATG by the MethPrimer program. C, the methylation levels of different regions of Oct4 and Thy1 in sperms, oocytes, PFFs and IVF blastocysts. According to the expression levels and methylation patterns of Oct4 and Thy1 in sperms, oocytes, PFFs and blastocysts, Region I of Oct4, including 16 CpG sites or Region II (10 CpG sites) of Thy1 could represent the methylation status of Oct4 or Thy1, respectively. Black or white circles indicate methylated or unmethylated CpG sites, respectively, and gray circles represent mutated and/or SNP variation at certain CpG sites. (+) represents gene expression, while (-) stands for no expression. The data are expressed as means ± SEM. a-dValues in the same group or at a given stage with different superscripts differ significantly (P<0.05). (TIF) [file pone.0129803.s001.tif]

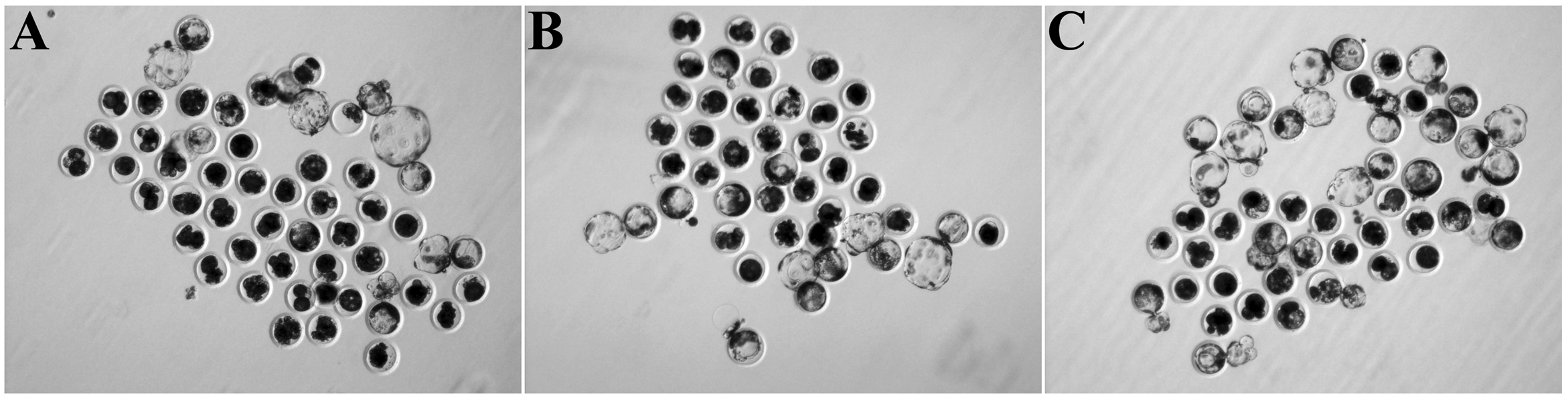

Supplement: S2 Fig — A, B and C, blastocysts (×40) derived from cloned embryos untreated, treated with 25 nM 5-aza-dC and treated with 40 nM TSA, respectively. (TIF) [file pone.0129803.s002.tif]

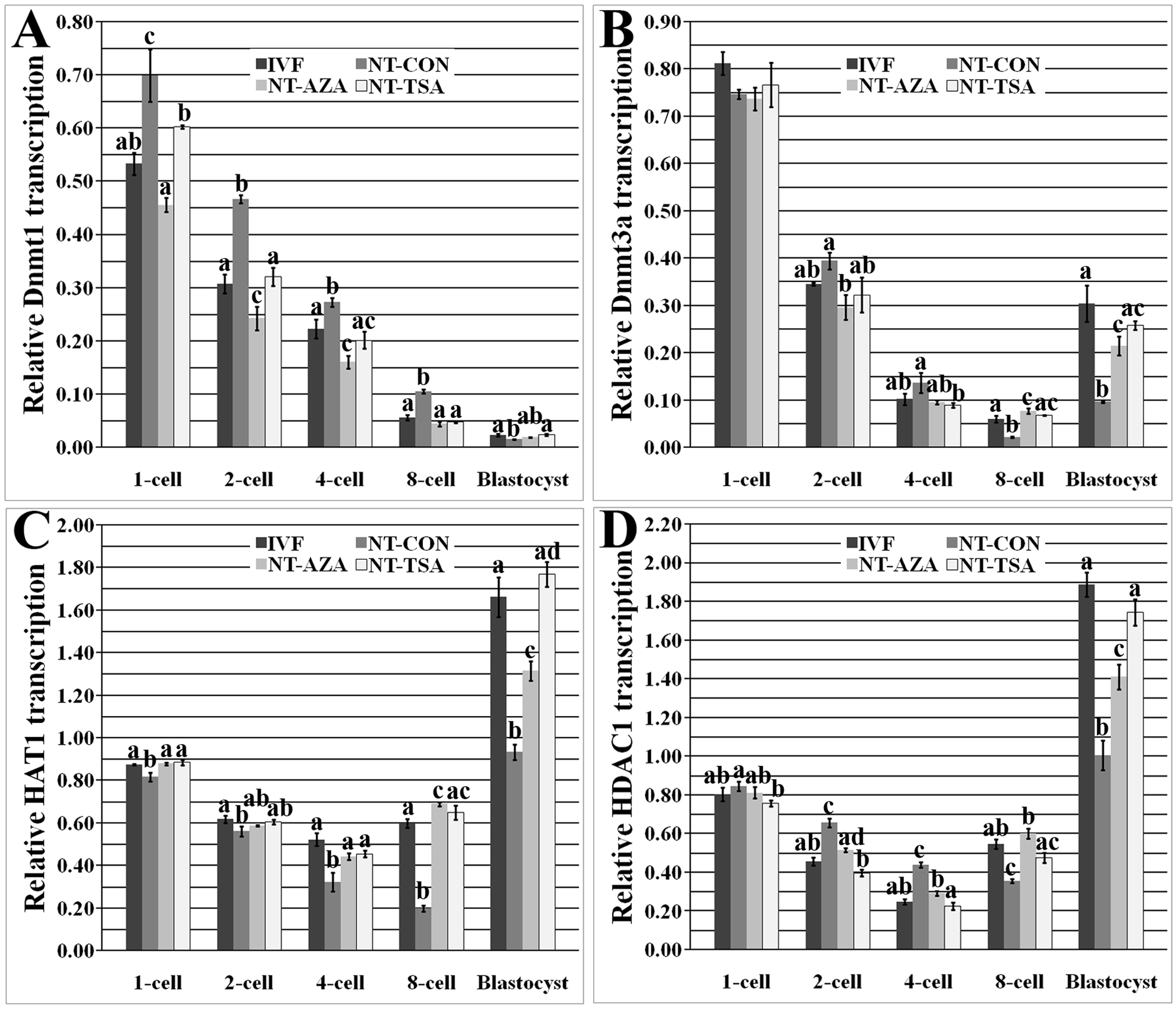

Supplement: S3 Fig — The expression patterns of Dnmt1 (A), Dnmt3a (B), Hat1 (C) and Hdac1 (D) at the 1-cell, 2-cell, 4-cell, 8-cell and blastocyst stages of IVF, NT-CON, NT-AZA and NT-TSA embryos. In comparison with IVF embryos, cloned embryos displayed the disrupted expression patterns of Dnmt1, Dnmt3a, Hat1 and Hdac1, while the expression profiles of these genes in NT-AZA or NT-TSA embryos were appropriately adjusted. The transcript abundance in MII oocytes was considered to be the control. The data were expressed as mean ± SEM. a-dValues with different superscripts differ significantly (P<0.05). (TIF) [file pone.0129803.s003.tif]

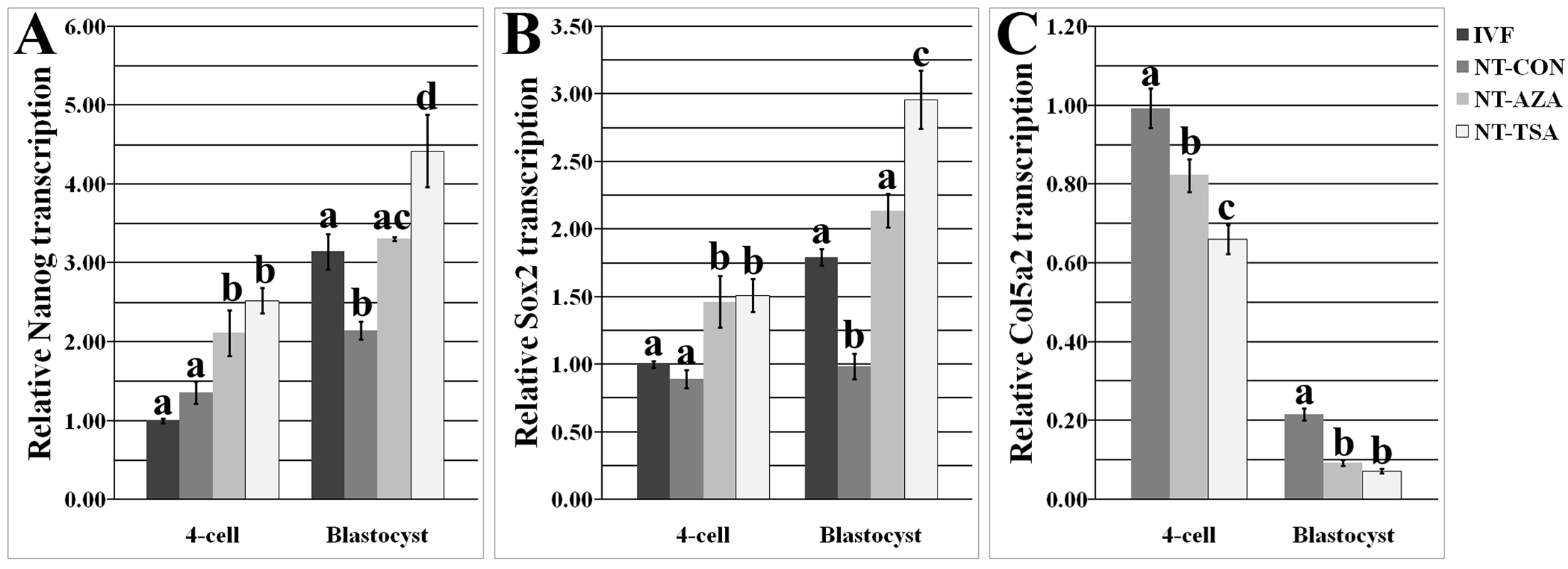

Supplement: S4 Fig — Relative transcripts of Nanog (A), Sox2 (B) and Col5a2 (C) at the 4-cell and blastocyst stages of IVF, NT-CON, NT-AZA and NT-TSA embryos. 5-aza-dC or TSA promoted the expression of Nanog, Sox2 and silenced the transcription of Col5a2 in cloned embryos. The transcript abundance in 4-cell cloned embryos was considered to be the control. The data were expressed as mean ± SEM. a-dValues at a given stage for the same gene with different superscripts differ significantly (P<0.05). (TIF) [file pone.0129803.s004.tif]

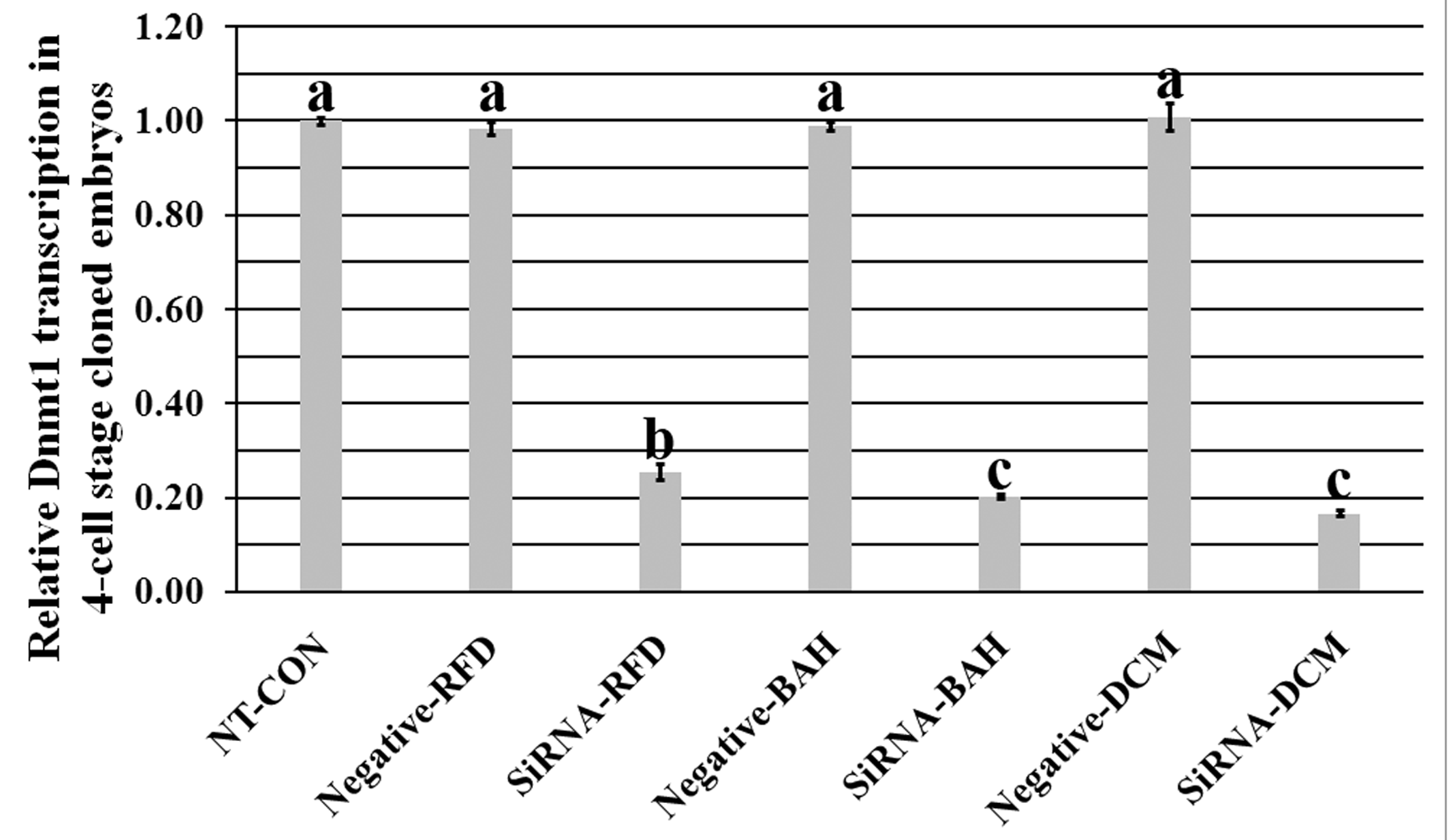

Supplement: S5 Fig — After siRNAs were injected into cloned embryos at 6 h post activation, the interference efficiency was measured in 4-cell cloned embryos. siRNA-RFD, siRNA-BAH and siRNA-DCM significantly reduced the expression of Dnmt1, and the interference efficiency of siRNA-DCM was the highest. The data were expressed as mean ± SEM. a-cValues with different superscripts differed significantly (P<0.05). (TIF) [file pone.0129803.s005.tif]

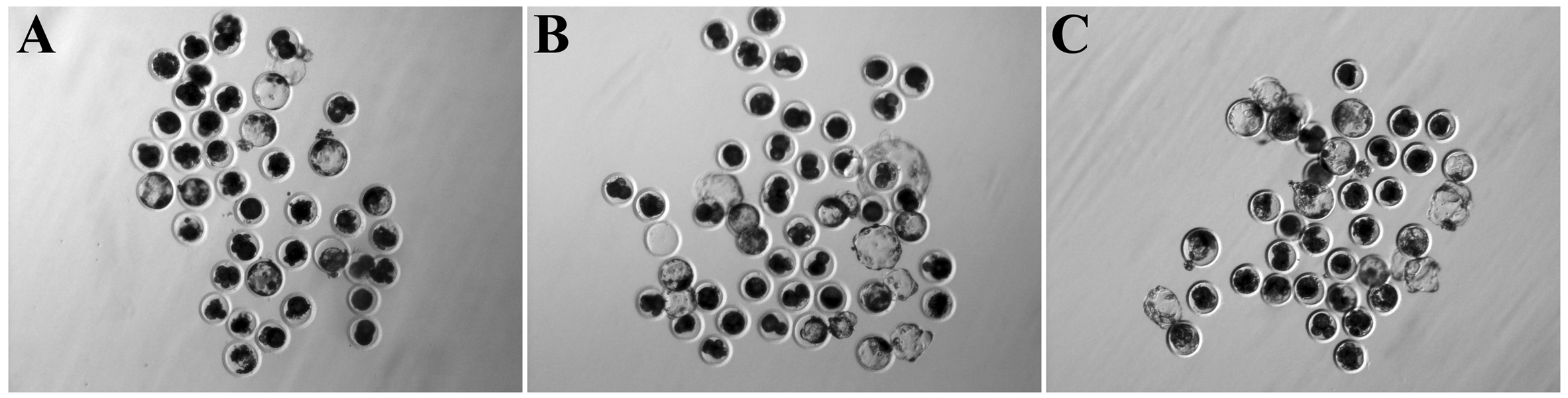

Supplement: S6 Fig — A, B and C, blastocysts (×40) derived from cloned embryos untreated, injected with negative siRNA and injected with siRNA. (TIF) [file pone.0129803.s006.tif]

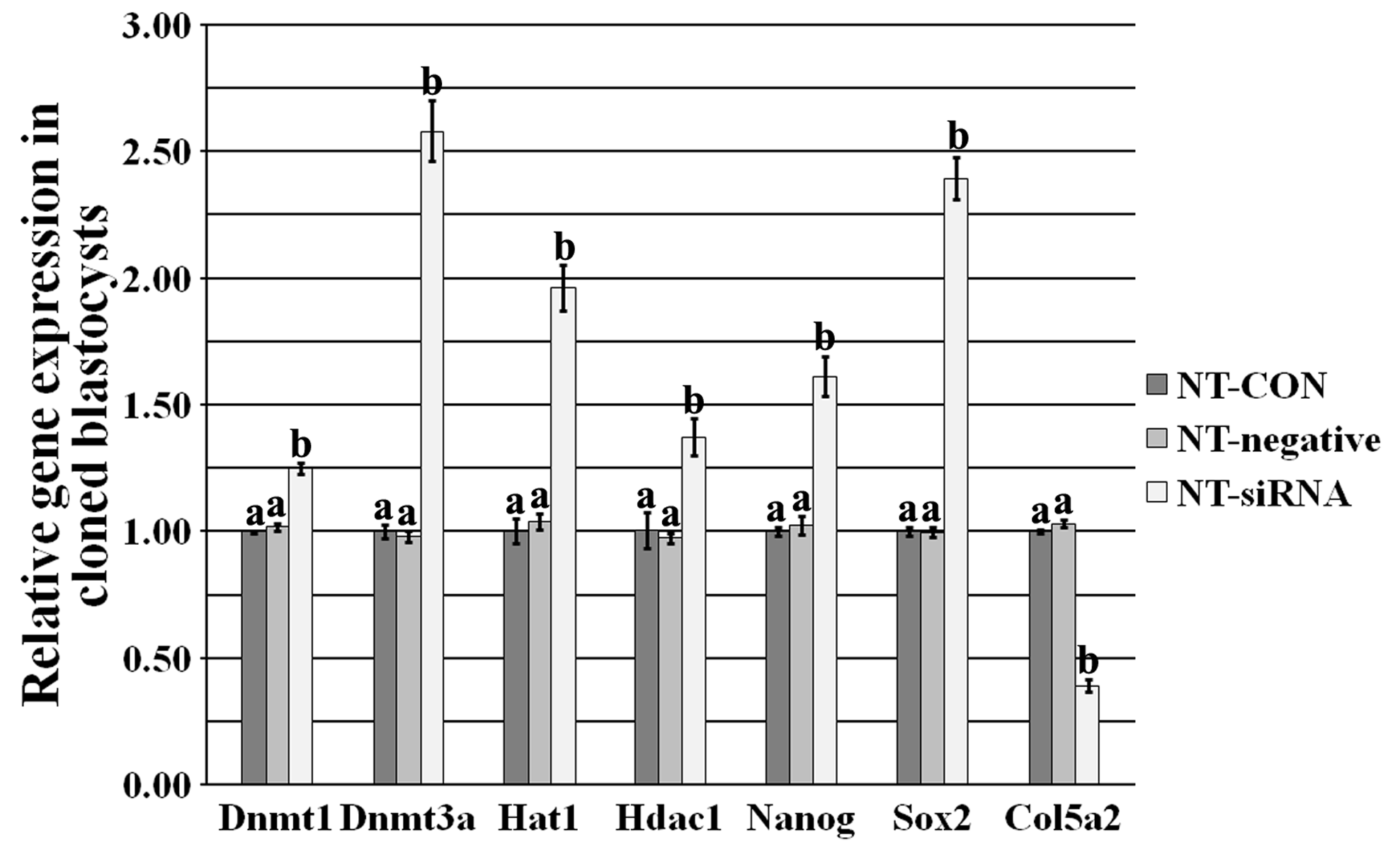

Supplement: S7 Fig — Compared with the NT-CON or NT-negative group, the NT-siRNA group displayed the upregulated expression of Dnmt1, Dnmt3a, Hat1, Hdac1, Nanog, Sox2 and downregulated transcription of Col5a2 in blastocysts. The transcript abundance of each gene in NT-CON embryos was considered to be the control. The data were expressed as mean ± SEM. a-bValues at a given stage for the same gene with different superscripts differ significantly (P<0.05). (TIF) [file pone.0129803.s007.tif]
