# Supplementary material for: Epigenetic Modification Agents Improve Gene-Specific Methylation Reprogramming in Porcine Cloned Embryos
Source: PLoS One. 2015 Jun 11;10(6):e0129803. doi: 10.1371/journal.pone.0129803 (PMC4465902; doi:10.1371/journal.pone.0129803)
Supplement: S1 Table — The primer sequence, amplified length and gene accession number for bisulfite sequencing and quantitative real-time PCR. (PDF) [file pone.0129803.s008.pdf]

Table S1 Details of primers for bisulfite sequencing and quantitative real-time PCR

Gene	Primer sequence (5'-3')	Length (bp)	Accession number
**Bisulfite sequencing**			
**Oct4 (Region I)**	Outer<br>F:ATTAGATTTGTGTGAGGATTTGAGAG<br>R:AAAACCCAATAAAACCAAAACTCTC	409	NC010449
	Inner<br>F:GAAGAGGGGTTTAATATTTGG<br>R:CCCAATCCCACCCACTAA	288	
**Oct4 (Region II)**	Outer<br>F:GGGGGATTTGTATTAAGGG<br>R:CTAAAACACCAACCCCACTC	529	NC010449
	Inner<br>F:GAGGTTAAGGTTAGTGGGTG<br>R:AACCCTTAAAAACTCAACCA	229	
**Thy1 (Region I)**	Outer<br>F:GTTAATTAAAATTAGATGTTAGTTTT<br>R:TCTACCCTATCTTATTTTTACAAC	320	NC010451
	Inner<br>F:GGTTTGGGAAGGAGTTGAT<br>R:AACACAAAACAAATAACCTAACC	202	
**Thy1 (Region II)**	Outer<br>F:GGTAGGGAGGTTATGGAAGTTA	497	NC_010451

	R:TAAATTCATTTACATTATAATACAAC<br>C		
	Inner<br>F:GGAAAGGTTTTGGTTTTAAGG<br>R:TCCAAAACTCTTAACATCCTACA	395	
**Thy1 (Region III)**	Outer<br>F:TATTGAGAGGTGGGAGGGGA<br>R:ACCCTCTCTCCCTTAATCACC	330	NC010451
	Inner<br>F:GTTTGGTAGTAAATGTGGGTA<br>R:CTTAATCACCCCTACCTTCTA	232	
**Quantitative real-time PCR**			
**Oct4**	F:GAAGGTGTTCAGCCAAACGAC<br>R:CGATACTTGTCCGCTTTC	185	NM001113060
**Thy1**	F:CCTCCATGGATCTGCTTATTC<br>R:CATCTGCTGGAGGCTGAGGT	209	NM001129971
**Sox2**	F:AACCAGAAGAACAGCCCAGAC<br>R:TCCGACAAAAGTTTCCACTCG	155	NM001123197
**Nanog**	F: CCTCCATGGATCTGCTTATTC<br>R: CATCTGCTGGAGGCTGAGGT	209	NM001129971
**Col5a2**	F:AGATGTACTTAAACAGGGACATTTG<br>GA<br>R:GTTATGGGGTCAGCACATTCAAGC	124	NM001105289
**Dnmt1**	F:GCGTCTTGCAGGCTGGTCAGTA<br>R:CTTCTTATCATCGACCACGACGCT	152	NM001032355

**Dnmt3a**	F:ATGTGGTTCGGAGACGGCAAGT<br>R:GCTCTCGTCGTTGTCATGGCA	195	NM001097437
**Hat1**	F: GACTGTGTGGAGGCAGATGATGTA<br>R:GAGAACCGAGTATGTATGGAGTAAGG	150	XM005671969
**Hdac1**	F: GCTGGCAAAGGCAAGTATTATG<br>R: CACACTGTAAGACGACCGCAC	139	XM005665200
**18s**	F:AATCTCGGGTGGCTGAACGC<br>R:CCGTTCTTAGTTGGTGGAGCGAT	143	NR002170
